# Energy balance and obesity: what are the main drivers?

**DOI:** 10.1007/s10552-017-0869-z

**Published:** 2017-02-17

**Authors:** Isabelle Romieu, Laure Dossus, Simón Barquera, Hervé M. Blottière, Paul W. Franks, Marc Gunter, Nahla Hwalla, Stephen D. Hursting, Michael Leitzmann, Barrie Margetts, Chizuru Nishida, Nancy Potischman, Jacob Seidell, Magdalena Stepien, Youfa Wang, Klaas Westerterp, Pattanee Winichagoon, Martin Wiseman, Walter C. Willett, On behalf of the IARC working group on Energy Balance and Obesity

**Affiliations:** 1 Nutrition and Metabolism Section, International Agency for Research on Cancer, 150 cours Albert Thomas, 69372 Lyon Cedex 08, France; 2 Centro de Investigación en Nutrición y Salud, Instituto Nacional de Salud Pública, Cuernavaca, Mexico; 3 Micalis Institute, MGP MetagenoPolis, INRA, AgroParisTech, Université Paris-Saclay, Jouy-en-Josas, France; 4 Genetic and Molecular Epidemiology Unit, Lund University Diabetes Centre, CRC, University hospital Malmö, Malmö, Sweden; 5 Faculty of Agricultural and Food Science, American University of Beirut, Beirut, Lebanon; 6 Department of Nutrition and the Nutrition Research Institute, The University of North Carolina, Chapel Hill, USA; 7 Department of Epidemiology and Preventive Medicine, University of Regensburg, Regensburg, Germany; 8 Faculty of Medicine, Southampton General Hospital, University of Southampton, Southampton, UK; 9 Nutrition Policy and Scientific Advice (NPU), Department of Nutrition for Health and Development (NHD), World Health Organization (WHO), Geneva, Switzerland; 10 Office of the Associate Director, Applied Research Program, Division of Cancer Control and Population Sciences, National Cancer Institute, Bethesda, USA; 11 Faculty of Earth and Life Sciences, Department of Health Sciences, University Amsterdam, Amsterdam, The Netherlands; 12 Department of Epidemiology and Environmental Health, School of Public Health and Health Professions, Joint Appointments, School of Medicine and Biomedical Sciences, University at Buffalo, State University of New York, Buffalo, USA; 13 NUTRIM School of Nutrition and Translational Research in Metabolism, Maastricht University, Maastricht, The Netherlands; 14 Institute of Nutrition, Mahidol University Salaya, Nakhon Pathom, Thailand; 15 World Cancer Research Fund International, London, UK; 16 Department of Nutrition, Harvard T.H. Chan School of Public Health, Boston, USA

**Keywords:** Energy intake, Energy expenditure, Energy balance, Obesity, Satiety, Diet

## Abstract

**Purpose:**

The aim of this paper is to review the evidence of the association between energy balance and obesity.

**Methods:**

In December 2015, the International Agency for Research on Cancer (IARC), Lyon, France convened a Working Group of international experts to review the evidence regarding energy balance and obesity, with a focus on Low and Middle Income Countries (LMIC).

**Results:**

The global epidemic of obesity and the double burden, in LMICs, of malnutrition (coexistence of undernutrition and overnutrition) are both related to poor quality diet and unbalanced energy intake. Dietary patterns consistent with a traditional Mediterranean diet and other measures of diet quality can contribute to long-term weight control. Limiting consumption of sugar-sweetened beverages has a particularly important role in weight control. Genetic factors alone cannot explain the global epidemic of obesity. However, genetic, epigenetic factors and the microbiota could influence individual responses to diet and physical activity.

**Conclusion:**

Energy intake that exceeds energy expenditure is the main driver of weight gain. The quality of the diet may exert its effect on energy balance through complex hormonal and neurological pathways that influence satiety and possibly through other mechanisms. The food environment, marketing of unhealthy foods and urbanization, and reduction in sedentary behaviors and physical activity play important roles. Most of the evidence comes from High Income Countries and more research is needed in LMICs.

## Introduction

Obesity is defined as a state of excess adiposity that presents a risk to health such as increased risk of chronic diseases including cancer [1–3] and is the consequence of sustained positive energy balance over time. Factors that influence energy balance can be considered as relating to the host (i.e., people), the environment (the set of external factors to which people are exposed) and the vector (food and drink). These factors interact in a complex way to influence eating and drinking patterns as well as activity behaviors. While experienced at the individual level, their roots lie in policies and actions that determine the environment, which may be local, national or international [4]. Therefore, understanding the relation between energy balance and obesity is a challenge and a necessity to develop effective prevention programs and policies. The International Agency for Research on Cancer (IARC) of the World Health Organization (WHO) convened a Working Group Meeting in December 2015 to review evidence regarding energy balance and obesity, with a focus on Low and Middle Income Countries (LMIC), and to tackle the following scientific questions:

1. Are the drivers of the obesity epidemic related only to energy excess and/or do specific foods or nutrients play a major role in this epidemic?
2. What are the factors that modulate these associations?
3. Which types of data and/or studies will further improve our understanding?

Each expert summarized the evidence from the literature on a specific topic in a written document that was reviewed by the IARC secretariat before the meeting and shared with the other participants (see list of topics in Table 1). Each topic was then briefly presented during the meeting and extensively discussed in plenary session with the other participants. A full report will be soon available on the IARC website. In this paper, we present a summary of the different topics that we addressed during the workshop and its conclusions and recommendations.

**Table 1:** List of topics presented during the working group meeting

Background introduction on obesity worldwide
Double burden malnutrition/obesity in LMICs
Can energy intake and expenditure (energy balance) be measured accurately in epidemiologic studies? Is this important?
How are components of dietary intake/dietary composition / foods/nutrients / related to obesity and weight gain?
How are overall energy intake and expenditure related to obesity?
Physical activity, sedentary behavior, and obesity
What existing epidemiologic data could serve to better understand the relation of energy intake and expenditure to obesity and the obesity epidemic?
Cultural determinants of obesity in low- and middle-income Countries of the Eastern Mediterranean Region
Potential mechanisms in childhood obesity
The interplay of genes, lifestyle and obesity (Presenter unable to attend, only written document)
Gut microbiota and obesity
Molecular and metabolic mechanisms underlying the obesity-cancer link
What steps should be recommended and implemented to prevent and control the obesity epidemic?
Which new data are needed to explore the relations of diet and dietary patterns to obesity and weight gain?

## Prevalence of overweight and obesity and double burden of malnutrition

**Fig. 1:**
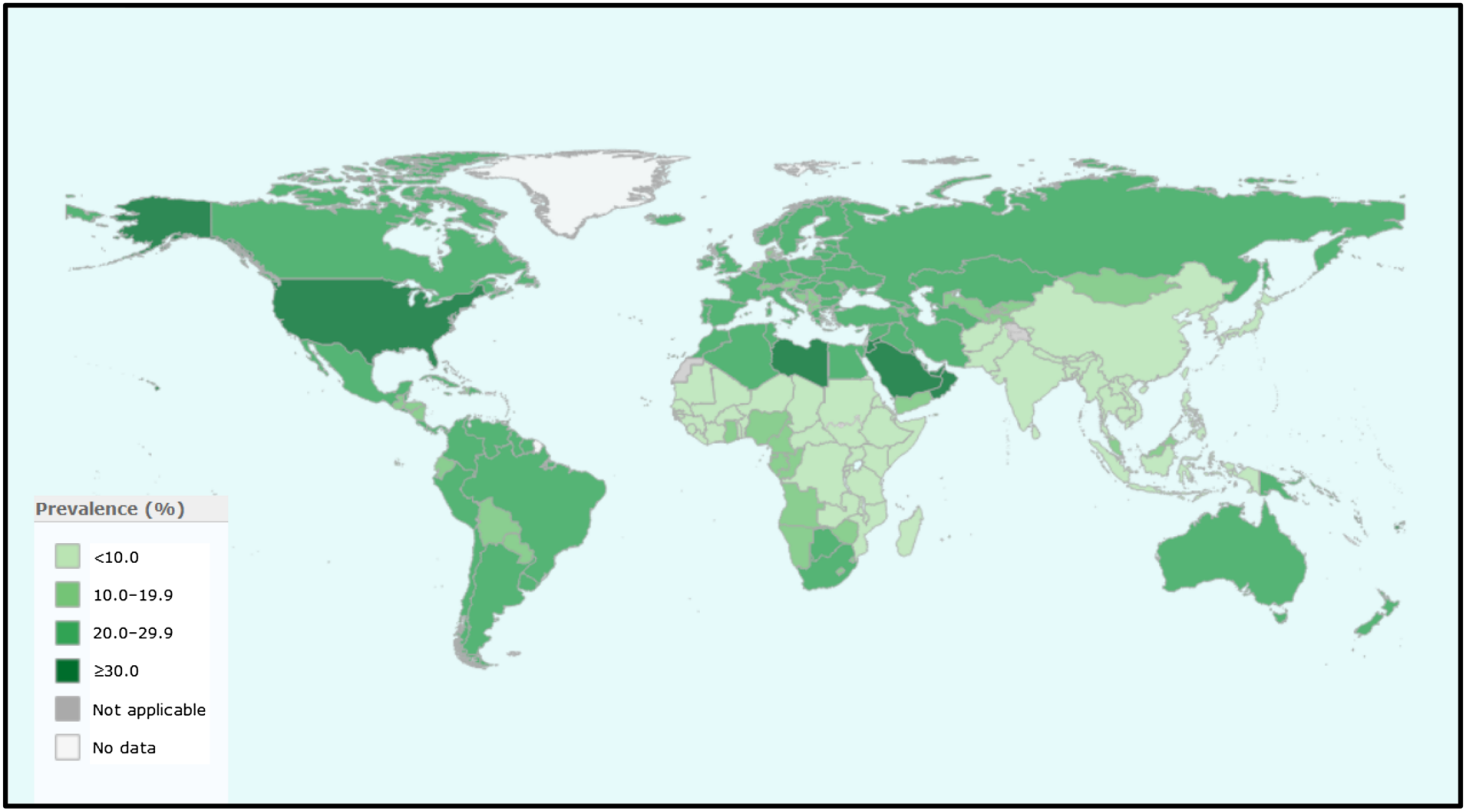
Prevalence of obesity, ages 18+, both sexes, 2014 (age-standardized estimates)—source: WHO

Obesity is now well recognized as a disease in its own right and overweight accounts for about 37% of the global burden of disease (2013) [5]. Obesity rates have been constantly increasing in the last thirty years with a worldwide prevalence that nearly doubled between 1980 and 2014 [2]. In 2014, 39% of adults aged 18 years and older (38% of men and 40% of women) were overweight [2]. In most parts of the world, women are more likely to be obese than men [2]. The prevalence of overweight and obesity tends to increase generally with the income level of the countries with the obesity prevalence in high-income and upper-middle-income countries being more than double that of low- income countries [2] (Fig. 1). In 2014, there were 41 million overweight children under age 5 years in the world; about 10 million more than two decades ago [5, 6]. Recent data indicate that the prevalence of childhood obesity in some developed countries (e.g., some European countries and the US) has apparently reached a plateau [7–9]. However, the number of overweight children in Low and Middle Income Countries (LMICs) has more than doubled since 1990, from 7.5 to 15.5 million [10]. In 2014, almost half of all overweight children under 5 lived in Asia and one quarter lived in Africa [5, 6].

In many LMICs, obesity coexists with undernutrition (including energy, and macro and micronutrient deficiencies) leading to a “double burden of malnutrition” (DBMN) occurring across the life-course of individuals and coexisting in the same households and communities/countries [11]. There is a complex interplay between early undernutrition (in mothers before and during pregnancy, and in early childhood) and later overnutrition that exacerbates the risk of non-communicable diseases that are rising rapidly in LMICs [12, 13].

Rapid economic growth and urbanization in LMICs have resulted in changing traditional dietary patterns, with increasing consumption of highly processed foods and beverages containing less nutrient dense diets, replacing or supplementing traditional plant-based diets, and simultaneous increase in sedentary behaviors and reduction of physical activity across all ages [14–17]. Soft drinks (sodas, sugar-sweetened beverages, SSB) consumption volumes have been increasing in almost all countries [18]. Between 1996 and 2002, sales of processed (packaged) foods grew by 28% in LMICS, compared with only 2.5% in HICs.

## Determinants of energy balance: what the evidence tells us

With recognition that overweight and obesity are major risk factors for cancer, cardiovascular disease, diabetes, and many other health conditions, the difference between energy intake and expenditure, frequently referred to as energy balance, has become of great interest because of its direct relation to long-term gain or loss of adipose tissue and alterations in metabolic pathways.

### Measurements of adiposity

Several measures for overweight and obesity have been used in epidemiological studies [3]; however, it is important to be aware that such measures are imperfect markers of the internal physiological processes that are the actual determinants of cancer development.

BMI (the quotient between weight in kilograms and height in meters squared) is the most commonly used body composition marker in epidemiologic studies due to its simplicity of assessment, high precision and accuracy, but it does not differentiate between lean and adipose tissue, or fat distribution, which varies across individuals, ethnicities, and throughout the lifespan. Nevertheless, BMI compares remarkably well to gold standard methods [24]. Waist circumference (WC) and waist-to-hip ratio (WHR) are useful to identify abdominal obesity but cannot clearly differentiate between visceral and subcutaneous fat compartments [25, 26]. Other measures that can be used in medium- or large-scale studies include skinfold thickness and bioelectrical impedance analysis, although the latter appears to add little to measures based on weight and height [27]. More direct measures of body composition are available, such as air displacement plethysmography, underwater weighing (hydrodensitometry), dual-energy X-ray absorptiometry, ultrasound, computed tomography and magnetic resonance imaging [28, 29]. Although reproducible and valid [30], these measures of body composition are, due to high costs and lack of portability, limited to small-scale studies that require a high level of accuracy. Their use in large-scale epidemiologic studies tends to be as reference methods [31].

### Energy intake and energy expenditure

Energy balance is the result of equilibrium between energy intake and energy expenditure. When energy intake exceeds expenditure, the excess energy is deposited as body tissue [1]. During adulthood, the maintenance of stable body weight depends on the energy derived from food and drink (energy intake) being equal to total energy expenditure over time. To lose body weight, energy expenditure must exceed intake, and to gain weight, energy intake must exceed expenditure [32]. Very small deviations from energy balance, on the order of 1–2% of daily energy intake, can result in large long-term changes in body weight (~20 kg) [33].

Measuring dietary intake and energy expenditure is a challenge in epidemiology. Energy intake, in particular, besides sometimes considerable measurement error in its assessment, can be subject to selective biases, such as the tendency of overweight and obese people to underestimate their intake [27]. While some objective measures exist for assessing energy expenditure or physical activity [34], such tools are not available for energy intake. Thus, assessment of energy balance by calculating the difference between intake and expenditure is not practically useful in large scale population studies. When comparing four different methods of dietary assessment, Willett and colleagues have shown that for all measures of total energy expenditure or intake within person variability was high relative to the 1 or 2% difference between intake and expenditure that can be extremely important (personal communication). Over time the best practical marker of positive or negative energy balance is change in the body weight which is readily measured with high precision even by self-report [27]. Since body weight change cannot distinguish between loss or gain of lean or fat mass, interpretation of weight change in an individual rests on assumptions about the nature of tissues lost or gained if body composition is not measured directly [35]. However, for most people, weight gain over a period of years during adulthood is largely driven by gain in fat mass.

In conclusion, body weight and change in weight provide precise indicators of long-term deviations in energy balance and are widely available for epidemiology studies. These simple and inexpensive measures of energy balance can be used both as exposure and outcome variables, taking into consideration their other determinants and confounding factors. Although not useful for assessing energy balance, which requires extreme accuracy and precision, measures of energy intake and physical activity will continue to play other important roles in epidemiologic studies and in monitoring population trends.

### Understanding nutritional determinants of obesity

Many factors relating to foods and beverages have been shown to influence amounts consumed or energy balance over the short to medium term, such as energy density and portion size [36, 37], although the effect of energy density over the longer term is unclear.

#### Foods and dietary patterns

One factor that has been suggested as being obesogenic is a high energy density of foods (i.e., an energy content of more than about 225–275 kcal per 100 g) [38]. However, there are exceptions; for example, nuts and olive oil (both extremely energy dense) did not increase weight when added to a diet [39]. Fast foods are energy-dense micronutrient-poor foods often high in saturated and trans fatty acids, processed starches and added sugars [40]. Thus, the extent that these foods are obesogenic may be related to their composition rather than to their energy density. Several observational studies indicated a higher risk of obesity and weight gain in consumers of fast foods than in the non-consumers [41–44]. A recent study from the European Prospective Investigation into Cancer and Nutrition (EPIC) study reported that a high plasma level of industrial trans fatty acids, interpreted as biomarkers of dietary exposure to industrially processed foods, was associated with the risk of weight gain, particularly in women [45]. Evidence supporting an obesogenic effect of sugary drinks is strong, with high relevance to children, especially in low-income socioeconomic groups (consumption of SSB is 93% higher in low-income children than in high income children) [46] and LMICs (a 1% rise in soft drink consumption was associated with an additional 3.4 overweight adults per 100 and 2.3 obese adults per 100 in LMICs) [47]. A meta-analysis of 22 cohort studies showed that each increment of sugary drink a day was associated with a 0.05–0.06 unit increase in BMI in children and 0.12–0.22 kg increased weight gain in adults [48]. Another meta-analysis of 5 cohort studies indicated a 55% (32–82%) higher risk of being overweight in children consuming sugary drinks daily [49]. Consumption of sweets and desserts characterized by high sugar content and energy density has been associated with weight gain [38], as well as red and processed meat (37% increased risk of obesity for consumption of higher quantities of red and processed meat) [50]. Conversely, higher consumption of legumes, wholegrain foods including cereals, non-starchy vegetables, and fruits (which have relatively low energy density) as well as nuts (with high energy density) have been associated with a lower risk of obesity and weight gain [38]. The content of fiber, satiating effect of fat, and low glycemic index in many of these foods may play an important role.

Results from three U.S. cohorts indicated that better diet quality, i.e., higher scores on alternate Mediterranean Diet (MD) score, the Alternate Health Eating Index (HEI)-2010, and the Dietary Approaches to Stop Hypertension were associated with lower weight gain during adult life [51]. This was in agreement with the results obtained from European cohorts using similar indexes [52, 53]. A systematic review found, in 13 of 21 studies, a negative association between adherence to Mediterranean diet (MD) and overweight/obesity or weight gain (i.e., 29% lower risk of being obese for men adhering to MD in a cohort study and up to 14 kg of weight lost after a 2-year MD intervention) [54].

Cohort studies conducted in LMICs would be valuable resources for understanding the impact of the nutrition and lifestyle transition on obesity. Some longitudinal studies have already been initiated in LMICs (as for instance the ones included in the Consortium of Health-Orientated Research in Transitioning Societies—COHORT [55], or the MTC cohort [56]). Building on these ongoing initiatives may prove informative and cost-efficient. Data from the Mexican Teacher cohort (MTC) have shown that women with a carbohydrates, sweet drinks and refined foods pattern were more at risk of having a larger silhouette and higher BMI, while a fruit and vegetable pattern was associated with a lower risk [57]. This emphasizes the need for public health interventions improving access to healthy diets, healthy food choices in the work place, and means of limiting consumption of beverages with a high sugar content and of highly processed foods, particularly those rich in refined starches.

#### Prevention of weight gain and/or maintenance of weight loss

Long-term experiments (>1 year) on prevention of weight gain suggest that the change in body fatness that occurs with modifying intakes seems to be mediated via changes in energy intakes, since isoenergetic exchange of sugars with other carbohydrates was not associated with weight change [49]. Evidence from randomized trials conducted in children and adolescents indicates that consumption of sugar-sweetened beverages, as compared with non-calorically sweetened beverages, results in greater weight gain and increases in the body mass index; however, the evidence is limited to a small number of studies [58, 59]. The findings of these trials suggest that there is inadequate energy compensation (degree of reduction in intake of other foods or drinks), for energy delivered as sugar dissolved in water [58].

#### Understanding weight loss

In weight loss trials, low carbohydrate interventions led to significantly greater weight loss than did low-fat interventions when the intensity of intervention was similar [60]. In a 2-year trial, where obese subjects were randomly assigned to low-fat restricted calorie, Mediterranean restricted-calorie or low-carbohydrate-restricted calorie diet, weight loss was similar in the MD and low-carb diet and significantly greater than in the low-fat diet. In their meta-analysis of 23 RCTs, Hu et al. [61] compared the effects of low-fat (≤30% energy) vs. low-carbohydrate (≤45% energy) diet and found that both types of diets resulted in comparable reductions in weight and waist circumference. However, compared with participants on low-fat diets, persons on low-carbohydrate diets experienced a slightly but statistically significantly lower reduction in total cholesterol and low-density lipoprotein cholesterol but a greater increase in high-density lipoprotein cholesterol and a greater decrease in triglycerides. The impact of reducing fat or carbohydrate may depend at least as much on the overall composition of the diet as on the reduction in the specific macronutrient targeted.

Most of these studies were conducted in HICs. This emphasizes the importance of conducting studies in LMICs in particular long-term dietary intervention trials focusing on alternative dietary patterns with foods readily available in these countries to propose viable changes in nutritional behaviors.

### Factors that modulate the association between dietary intake and obesity

#### Physical activity

Long-term observational studies fairly consistently show an association between physical activity and weight maintenance, and a 2009 position paper from the American College of Sports Medicine (ACSM) stated that 150–250 min per week of moderate intensity physical activity is effective to prevent weight gain [62]. The long-term effect of physical activity on weight loss has been less convincing and isolated aerobic exercise was not shown to be an effective weight loss therapy but may be effective in conjunction with diet [63]. Evidence suggests that diet combined with physical activity results in greater weight loss than diet alone and is more effective for increasing fat mass loss and preserving lean body mass and, therefore, it leads to a more desirable effect on overall body composition [64]. Weight maintenance after weight loss is improved with physical activity and >250 min of physical activity per week has been recommended [62]. Intervention studies have consistently found no effect of resistance exercise on reducing body weight [62] or visceral adipose tissue [65]. However, resistance training appears to be more effective in increasing lean body mass than aerobic training and the combination of aerobic and resistance training may be the most efficient exercise training modality for weight loss [66]. In recent years, physical activity research has expanded its focus to include the potentially detrimental effects of sedentary behavior on energy balance. Findings from the Nurses’ Health Study showed that time spent watching television viewing was positively related to risk of obesity (each 2-h/d increment in TV watching was associated with a 23% increase in obesity) [67]. Sedentary behavior also represents an independent risk factor for obesity in children and adolescents [68]. In short-term studies, higher levels of physical activity have been shown to mitigate the effect of increasing energy density on weight gain, and it appears that at the low levels of physical activity typical of current high income populations, adequate suppression of appetite to maintain energy balance is compromised [69].

In conclusion, moderate intensity physical activity performed for 150–250 min per week appears to prevent weight gain and produces modest weight loss in adults. Greater amounts of moderate intensity physical activity (>250 min per week) are required for weight maintenance following weight loss. Resistance exercise does not appear to decrease body weight or body fat but it promotes gain of lean body mass, and the combination of resistance and aerobic exercise seems to be optimal for weight loss. Physical activity improves chronic disease risk factors independent of its impact on body weight regulation. Moreover, sedentary behavior represents an independent risk factor for the development of overweight and obesity.

#### Genetic and epigenetic factors

The patterns and distributions of obesity within and between ethnically diverse populations living in similar and contrasting environments suggest that some ethnic groups are more susceptible than others to obesity [70]. More than 150 common genetic variants have been robustly associated with measures of body composition [71], though the individual impact of each variant is small. There is now convincing epidemiological evidence of interactions between common variants in the *FTO* (Fat mass and obesity-associated protein) gene and lifestyle with respect to obesity [72–74]. However, almost all these data are from cross-sectional studies, and temporal relationships are not clear. There are large studies supporting gene–lifestyle interactions at several other common loci, but the burden of evidence is far less for these loci than for *FTO* [75, 76]. However, the magnitude of the interaction effects reported for *FTO* (or other common variants) is insufficient to warrant the use of those data for clinical translation. Potentially reversible epigenetic changes in particular altered DNA methylation patterns could also serve as biomarkers of energy balance and mediators of gene–environment interaction in obesity [77]. Several ‘epigenome-wide association studies’ have now been conducted and have identified a panel of gene loci where methylation levels significantly differ in obese and lean individuals [78, 79]. Such discoveries could provide novel insights into how energy balance and its determinants influence obesity development, interaction with diet and environmental factors and subsequent metabolic dysregulation.

In summary, there is an abundance of published evidence, predominantly from cross-sectional epidemiological studies, that supports the notion that lifestyle and genetic factors interact to cause obesity. However, few studies have been adequately replicated, and functional validation and specifically designed intervention studies are rarely undertaken, both of which are necessary to determine whether observations of gene–lifestyle interaction in obesity are causal and of clinical relevance.

#### Microbiota

In a healthy symbiotic state, the colonic microbiota interacts with our food, in particular dietary fiber, allowing energy harvest from indigestible dietary compounds. It also interacts with cells, including immune cells, as well as with the metabolic and nervous systems; and protects against pathogens. Conversely, a dysbiotic state is often associated with diseases including not only inflammatory bowel diseases (IBD), allergy, colorectal cancer and liver diseases, but also obesity, diabetes and cardiovascular diseases [80]. Dysbiosis may be defined as an imbalanced microbiota including loss of keystone species, reduced richness or diversity, increased pathogens or pathobionts or modification or shift in metabolic capacities [81]. Dysbiosis in the intestinal microbiota has been associated with obesity [82]. A loss of bacterial gene richness is linked to more severe metabolic syndrome, and less sensitivity to weight loss following caloric restriction diet [83]. Dietary habits also seem to be associated with microbiota richness [84]. The proposed mechanisms by which gut microbiota dysbiosis and loss of richness can promote obesity and insulin resistance are diverse, often derived from mouse models, and still deserve more studies and validation in humans.

#### Determinants of childhood obesity

Many factors have contributed to the increase in the prevalence of obesity in children including unhealthy dietary patterns with high consumption of fast foods and highly processed food [85], of sugar sweetened beverages [86], lack of PA, an increase in sedentary behaviors (e.g., screen time) [87], and shorter sleep duration [88]. Other factors, such as changes in parenting and family factors, school factors, social norms, community food and PA environments that affect children’s eating and PA have also contributed [89, 90]. Experiences during early life (e.g., prenatal factors such as the in utero exposures experienced and postnatal factors such as infant and young child feeding) can have an important, long-term impact on future health, including obesity risk [91]. In particular, maternal gestational weight gain (GWG) [92], maternal overweight prior to pregnancy, smoking during pregnancy, high or low infant birth weight, rapid weight gain during the first year of life [93–95], early obesity rebound [96], breastfeeding patterns [97] and early introduction of complementary food [98] have all been linked to later excess adiposity. Many of these are inter-related and work is ongoing to disentangle concurrent factors. In addition, high levels of stress during childhood and adolescence may change eating habits and augment consumption of highly palatable but nutrient-poor foods [99].

Multiple factors at the individual, family, school, society and global levels impact children’s energy-balance-related behaviors, and have contributed to the increases in childhood obesity worldwide. Although genetic factors may play a role in affecting individuals’ susceptibility of developing obesity, environmental factors should be the key targets of intervention efforts to fight the epidemic as they are modifiable.

## Prevention of obesity

Numerous policy options to prevent obesity have been explored, and evidence is sufficient to conclude that many are cost effective. Given the multifactorial nature of obesity, as in other complex public health problems, a combination of interventions is more likely to generate better results than focusing only on a single measure [100]. Gortmaker et al. [101] estimated the cost-effectiveness of seven interventions that are generally considered to be most promising. They modeled the reach, costs and savings for the US population 2015-25. Some of these interventions (excise tax on sugar-sweetened beverages, elimination of tax deduction for advertising unhealthy food to children and nutrition standards for food and beverages sold in schools outside of meals) not only prevent many cases of childhood obesity, but also potentially cost less to implement than they would save for society.

The global childhood obesity epidemic demands a population-based multisector, multi-disciplinary, and culturally relevant approach. Children need protection from exploitative marketing and special efforts to support healthy eating, PA behaviors, and optimal body weight [102–104]. Adequate evidence has been accumulated that interventions, especially school-based programs, can be effective in preventing childhood obesity [105]. Preventing obesity will require sustained efforts across all levels of government and civil society.

Although there are individual differences in susceptibility, obesity is by large a societal problem resulting from health related behaviors that are largely driven by environmental upstream factors. Many options for policies to prevent obesity are available and many of these are effective and cost-effective. Integrated management of the epidemic of obesity requires top-down government policies and bottom-up community approaches and involvement of many sectors of society. Integrating evidence-based prevention and management of obesity is essential.

## The obesity-cancer link: what are the underlying mechanisms?

There is convincing evidence for a role of obesity as a causal factor for many types of cancer including colorectum, endometrium, kidney, oesophagus, postmenopausal breast, gallbladder, pancreas, gastric cardia, liver, ovary, thyroid, meningioma, multiple myeloma, and advanced prostate cancers [19]. Recent progress on elucidating the mechanisms underlying the obesity-cancer connection suggests that obesity exerts pleomorphic effects on pathways related to tumor development and progression and, thus, there are potential opportunities for primary to tertiary prevention of obesity-related cancers. We now know that obesity can impact well-established hallmarks of cancer (such as genomic instability, angiogenesis, tumor invasion and metastasis and immune surveillance) [20]. However, obesity-associated perturbations in systemic metabolism and inflammation, and the interactions of these perturbations with cancer cell energetics, are emerging as the primary drivers of obesity-associated cancer development and progression. In both obesity and metabolic syndrome, alterations occur in circulating levels of insulin and insulin-like growth factors, sex hormones, adipokines, inflammatory factors, several chemokines, lipid mediators and vascular associated factors [21–23].

## Which types of data and /or studies will further improve our understanding?

Most research on obesity and cancer has focused on Caucasians in HICs. While many of the identified risk factors in HICs will have the same physiologic effects in LMICs, the determinants may be different, in addition to other environmental and genetic differences across populations. Novel risk factors or traditional diets may be identified in newly studied populations and regions. Diet is shaped by many factors such as traditions, knowledge about diet, food availability, food prices, cultural acceptance, and health conditions. Likewise, a variety of factors will influence daily physical activity and sedentary behaviors, including dwellings, urbanization, opportunities for safe transportation by bicycle riding and walking, recreational facilities, employment constraints and health conditions. Surveillance of current diet and health conditions and assessment of trends over time is of major importance in LMICS. Further resources and research capacity are of highest priority.

In addition to surveillance efforts, prospective studies able to document lifestyle and change of lifestyle over time are an important area of research. Several cohort studies conducted in HICs have shown an impact of healthy dietary patterns on obesity [106] and similar studies could be conducted in LMICs to identify dietary patterns related to weight gain and obesity in a variety of settings to evaluate the major lifestyle, behavioral and policy influences in an effort to plan public health interventions appropriately. Intervention and implementation research in different countries/regions is needed to learn about social and physiological changes and the sustainability of the changes, in particular among children and adolescents in both school-based and non-school-based setting as well as research on the cost effectiveness of policies.

A major challenge is to capture life course exposures and identify windows of susceptibility. Early exposure to poor diet and sugar-sweetened beverages, sedentariness, tobacco smoke and other environmental exposures can alter infants’ and children’s growth patterns and may result in altered metabolism, obesity, and risk of chronic disease in adulthood [107]. Cohort studies covering the whole life course, focusing on critical windows of exposure and the time course of exposure to disease (birth cohorts, adolescent cohorts, and young adult cohorts), should be considered. Of particular interest are multi-centered cohorts and inter-generational cohorts that would create resources to enable research on the interplay between genetics, lifestyle and the environment. For example in the Avon longitudinal study of parents and children (ALSPAC), increasing intake of energy-dense nutrient-poor foods during childhood (mostly free sugar) was associated with obesity development. Diets with higher energy density were associated with increased fat mass [108]. Most relevant to LMICs is the observation that children who were stunted in infancy and are subsequently exposed to more calories, at puberty, are more likely to have higher fat mass at the same BMI compared with children who were not stunted [93, 94, 109]. Poor maternal prenatal dietary intakes of energy, protein and micronutrients have been associated with increased risk of adult obesity in offspring while a high protein diet during the first 2 years of life was also associated with increased obesity later in life [110]; conversely, exclusive breastfeeding was associated with lower risk of obesity later in childhood, although this may not persist into adulthood [111]. Similar results from a cohort study conducted in Mexico show that children exclusively or predominantly breastfed for 3 months or more had lower adiposity at 4 years [112]. Further work on birth cohorts or other prospective studies in LMICs is likely to provide insights into the developmental causes of obesity and NCDs. Input from local research communities, health ministries and policy makers and appropriate funding or resource assignment are critical for the success of new efforts in LMICs.

There is clearly a need for capacity building and resources devoted to nutritional research in LMICs. The first step would be a comprehensive assessment of resources already in place, and the identification of gaps and priorities for moving forward. Repeated surveillance surveys are essential in LMICs for evaluation of current and future status of the population and addressing undesirable trends with prevention and control programs. It is recognized that few prospective studies are currently underway in LMICs and resources will be needed to pursue this important area of research. Input from local research communities, health ministries and policy makers are critical for the success of new efforts in LMICs.

## Conclusions and recommendations

The global epidemic of obesity and the double burden of malnutrition are both related to poor quality diet; therefore, improvement in diet quality can address both phenomena.

The benefits of a healthy diet on adiposity are likely mediated by effects of dietary quality on energy intake, which is the main driver of weight gain. Energy balance is best assessed by changes in weight or in fat mass. Measures of energy intake and expenditure are not precise enough to capture small differences that are of individual and public health importance. The quality of the diet may exert its effect on energy balance through complex hormonal and neurological pathways that influence satiety and possibly through other mechanisms.

Dietary patterns characterized by higher intakes of fruits and vegetables, legumes, whole grains, nuts and seeds and unsaturated fat, and lower intakes of refined starch, red meat, trans and saturated fat, and sugar-sweetened foods and beverages, consistent with a traditional Mediterranean diet and other measures of dietary quality, can contribute to long-term weight control. Limiting consumption of sugar-sweetened beverages has a particularly important role in weight control. In weight loss trials, existing evidence does not support the role of the reduction in the percentage of energy from fat on weight loss, though the reductions in fat may not have been low enough for the outcome and the effects of single macronutrients cannot be adequately captured without specifying replacement/comparison sources of energy.

Genetic factors cannot explain the global epidemic of obesity. It is possible that factors such as genetic, epigenetic and the microbiota can influence individual responses to diet and physical activity. Very few gene–diet interactions or diet-microbiota have been established in relation to obesity and effects on cancer risk.

Short-term studies have not provided clear benefit of physical activity for weight control, but meta-analysis of longer term trials indicates a modest benefit on body weight loss and maintenance. The combination of aerobic and resistance training seems to be optimal. Long-term epidemiologic studies also support modest benefits of physical activity on body weight. This includes benefits of walking and bicycle riding, which can be incorporated into daily life and be sustainable for the whole population. Physical activity also has important benefit on health outcomes independent of its effect on body weight. In addition, long-term epidemiologic studies show that sedentary behavior (in particular TV viewing) is related to increased risk of obesity, suggesting that limiting sedentary time has potential for prevention of weight gain.

The major drivers of the obesity epidemic are the food environment, marketing of unhealthy foods and beverages, urbanization, and probably reduction in physical activity. Existing evidence on the relations of diet, physical activity and socio-economic and cultural factors to body weight is largely from HICs. There is an important lack of data on diet, physical activity and adiposity in most parts of the world and this information should to be collected in a standardized manner when possible. In most environments, 24h recalls will be the more suitable method for dietary surveillance. Attention should be given to data in subgroups because mean values may obscure important disparities. In utero and early childhood, environment has important implications for lifetime adiposity. This offers important windows of opportunity for intervention. Observational data on determinants of body weight and intervention trials across the life course to improve body weight are also required. To accomplish these goals, there is a need for resources to build capacity and conduct translational research.

Gaining control of the obesity epidemic will require the engagement of many sectors including education, healthcare, the media, worksites, agriculture, the food industry, urban planning, transportation, parks and recreation, and governments from local to national. This provides the opportunity for all individuals to participate in this effort, whether at home or in establishing high-level policy. We now have evidence that intensive multi-sector efforts can arrest and partially reverse the rise of obesity in particular among children. In conclusion, we are gaining understanding on the determinants of energy balance and obesity and some of these findings are being translated into public health policy changes. However, further research and more action from policy makers are needed.
